# Inversion of the Balance between Hydrophobic and Hydrogen Bonding Interactions in Protein Folding and Aggregation

**DOI:** 10.1371/journal.pcbi.1002169

**Published:** 2011-10-13

**Authors:** Anthony W. Fitzpatrick, Tuomas P. J. Knowles, Christopher A. Waudby, Michele Vendruscolo, Christopher M. Dobson

**Affiliations:** Department of Chemistry, University of Cambridge, Cambridge, United Kingdom; Stanford University, United States of America

## Abstract

Identifying the forces that drive proteins to misfold and aggregate, rather than to fold into their functional states, is fundamental to our understanding of living systems and to our ability to combat protein deposition disorders such as Alzheimer's disease and the spongiform encephalopathies. We report here the finding that the balance between hydrophobic and hydrogen bonding interactions is different for proteins in the processes of folding to their native states and misfolding to the alternative amyloid structures. We find that the minima of the protein free energy landscape for folding and misfolding tend to be respectively dominated by hydrophobic and by hydrogen bonding interactions. These results characterise the nature of the interactions that determine the competition between folding and misfolding of proteins by revealing that the stability of native proteins is primarily determined by hydrophobic interactions between side-chains, while the stability of amyloid fibrils depends more on backbone intermolecular hydrogen bonding interactions.

## Author Summary

In order to carry out their biological functions, most proteins fold into well-defined conformations known as native states. Failure to fold, or to remain folded correctly, may result in misfolding and aggregation, which are processes associated with a wide range of highly debilitating, and so far incurable, human conditions that include Alzheimer's and Parkinson's diseases and type II diabetes. In our work we investigate the nature of the fundamental interactions that are responsible for the folding and misfolding behaviour of proteins, finding that interactions between protein side-chains play a major role in stabilising native states, whilst backbone hydrogen bonding interactions are key in determining the stability of amyloid fibrils.

## Introduction

Defining the rules of protein folding, a process by which a sequence of amino acids self-assembles into a specific functional conformation, is one of the great challenges in molecular biology [1]–[3]. In addition, deciphering the causes of misfolding, which can often result in the formation of 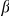-sheet rich aggregates, is crucial for understanding the molecular origin of highly debilitating conditions such as Alzheimer's and Parkinson's diseases and type II diabetes [4].

**Figure 1:**
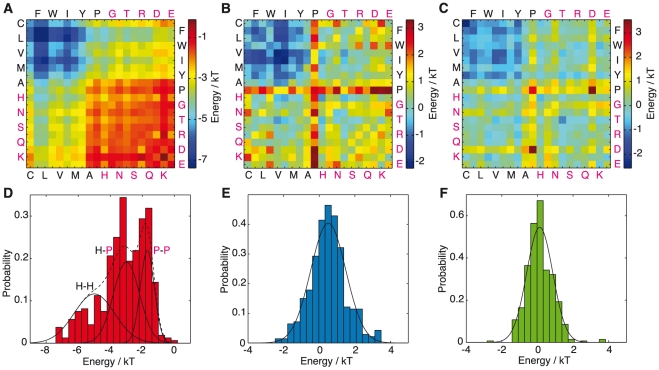
PDB-derived statistical potentials for folding to the native state [**5**] and to 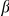-sheet rich (amyloid-like) states [9]. (A–C) Plots of the elements of the MJ matrix (A), the parallel (B) and antiparallel (C) matrices. Hydrophobic residues are shown in black and hydrophilic residues in magenta. (D) Distribution of free energies in the MJ matrix showing the decomposition of contacts into hydrophobic-hydrophobic (H-H, 37% of all contacts, −4.99 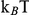, s.d. 1.27 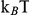), hydrophobic-polar (H-P, 39% of all contacts, −2.99 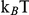, s.d. 0.82 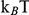) and polar-polar (P-P, 24% of all contacts, −1.69 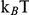, s.d. 0.44 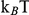). The sum of these components is shown as a dashed line. (E,F) Single Gaussian fits to the distributions of parallel (E) and antiparallel (F) contact free energies (0.51, s.d. 0.99 and 0.13, s.d. 0.73 (in 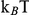) respectively).

Major advances in establishing the interactions that drive the folding process have been made by analysing the structures in the Protein Data Bank (PDB), and particularly by examining the frequency with which contacts between the different types of amino acid residues occur [5]. In this statistical approach, interaction free energies are derived from the probability, 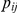, of two amino acids of types 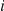 and 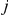 being in contact in a representative set of protein structures using the Boltzmann relation 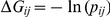. This operation defines a 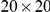 matrix that lists the free energies of interaction between amino acid pairs. One of the most studied matrices of this type has been reported by Miyazawa and Jernigan [5]. Three distinct analyses of this matrix (Fig. 1A) have all revealed that residue-water interactions play a dominant role in protein folding [6]–[8].

More recently, the same statistical potential method has been used to investigate aggregation of soluble proteins into the amyloid state, now recognised as a generic, alternative, stable and highly organised type of protein structure [3]. A method for predicting the stability of amyloid structure (PASTA) [9] extracts the propensities (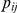) of two residues found on neighbouring strands in parallel or antiparallel 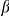-sheets in a representative set of PDB structures. The resulting 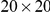 parallel strand and antiparallel strand interaction free energy matrices (referred to here as “parallel” and “antiparallel” respectively) are shown in Fig. 1B and 1C. Owing to the absence of a large number of solved atomic resolution amyloid fibril structures in the PDB, the central assumption of the PASTA approach is that the side-chain interactions found in the 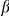-sheets of globular proteins are the same as those stabilising 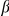-sheets in the core of amyloid fibrils [9]. This assumption is supported by the observation that the PASTA matrices are highly successful at predicting the portions of a polypeptide sequence that stabilise the core regions of experimentally determined amyloid fibrils and the intra-sheet registry of the 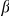-sheets [9]. We therefore treat the PASTA matrices as statistical potentials for the parallel and antiparallel 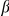-sheets found in the core of amyloid fibrils [9].

In this work we carry out a comparative analysis of the interaction matrices for folding and amyloid formation, in order to reveal the nature of the interactions that drive these two processes, and to provide fundamental insight into the competition between them. Our results indicate that the balance between hydrophobic and hydrogen bonding interactions is inverted in these two processes.

## Results

### Analysis of interaction free energy matrices

The contact approximation for the effective Hamiltonian, 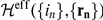, used to describe a system of polypeptide chains usually takes the form
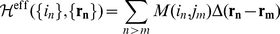
(1)where 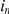 is the residue type 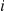 at position 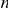 along the polypeptide chain, 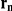 is the position of residue 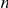 and 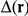 is a function reflecting the fact that two amino acids interact with free energy 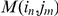 when they are in spatial proximity to each other [10].

For random heteropolymers, the pairwise contact free energies 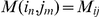 can be approximated as a set 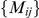 of 210 independent random variables (i.e. the 210 independent elements in a 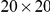 symmetric matrix). For the MJ matrix, a plot with the axes running from hydrophobic (C,F,L,W,V,I,M,Y,A,P, black) [11] to hydrophilic (H,G,N,T,S,R,Q,D,K,E, magenta) [11] residue types reveals three large blocks of hydrophobic interactions (Fig. 1A). The most stabilising interactions are hydrophobic-hydrophobic (Fig. 1A, top left corner, blue), followed by hydrophobic-polar (Fig. 1A, bottom left corner and top right corner, yellow/green) and polar-polar interactions (Fig. 1A, bottom right corner, red).

On closer inspection, analysis of these interactions in the form of a histogram shows that the distribution of contact free energies determined from the Miyazawa-Jernigan (MJ) matrix (Fig. 1D) can be represented as the sum of three Gaussian terms corresponding to hydrophobic-hydrophobic (H-H), hydrophobic-polar (H-P) and polar-polar (P-P) contacts [6] (Fig. 1D). This interpretation implies that globular proteins are stabilised mainly by side-chain hydrophobic interactions [6] since the sum of all H-H, H-P and P-P contacts captures the overall distribution of contact free energies extremely well (Fig. 1D).

In contrast to the MJ matrix, contour maps of the parallel and antiparallel 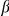-sheet contact matrices of the type characteristic of amyloid fibrils [4] show highly destabilising contact free energies between all Pro-X pairs (Fig. 1B, C, proline row, proline column, red/yellow). Since proline cannot form inter-molecular backbone hydrogen bonds this observation suggests that the stabilisation of 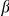-sheets arises mainly from the dominance of backbone hydrogen bonding, with hydrophobic interactions (Fig. 1B, C, top left corner, blue) playing a secondary role. Furthermore, plots showing the distribution of the contact free energies from parallel and antiparallel 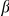-sheets (Fig. 1E, F) of the type found in amyloid structures [4] indicate, unlike the situation for native folds described above, a single narrow Gaussian distribution for polar and non-polar contacts alike. This result, combined with the significance of the destabilising Pro-X contacts, is consistent with the view that a major role in protein aggregation into amyloid fibrils is played by backbone hydrogen bonding interactions [12]–[14], which are “generic” [3] to any polypeptide chain, although sequence-dependent effects are also important to modulate the propensity of specific peptides and proteins [15]–[17].

The difference in these probability distributions arises because we are examining the contact free energies that define the protein folding and misfolding free energy minima *via* the MJ and PASTA matrices respectively. It is clear that the possible number of ways of forming a given contact between amino acids 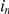 and 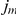 is greater in globular proteins than in fibrillar aggregates as the area of Ramachandran space available to 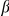-sheets (13.3% of the total 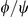 space) is much smaller than that accessible to native proteins. In addition, the type of amino acid and specific sequence patterns have varying degrees of globularity [18] or aggregation propensity [16] with certain amino acids, notably proline, appearing much more frequently in globular proteins than in the core region of amyloid fibrils [9].

To investigate the consequences of these differences in the conformational spaces relevant to folding and misfolding we consider the constrained sampling of the protein Hamiltonian 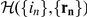 over a subspace 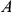 of conformational space, which is given formally by
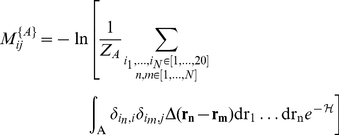
(2)where 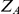 is the partition function sampled over the subspace 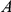. Interaction parameters to describe the folding process are usually defined by considering a subspace 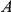 that includes the regions of conformational space corresponding to the native states of globular proteins [19]. By contrast, interaction parameters to describe the aggregation process are defined for a subspace 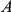 that includes only the regions of conformational space corresponding to 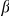-sheet rich structures such as 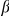-helices or amyloid fibrils [19]. While the Hamiltonian, 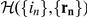, is invariant, the space over which it is integrated will vary depending on the region of conformational space that is being explored. In our case, this leads to distinct “effective” Hamiltonians for the protein folding and misfolding minima; these Hamiltonians have the same general form as Eq. [1] but have different amino acid interaction matrices 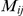, according to Eq. [2], depending on which process is involved. We thus conclude that there could be differences in the various effective energy terms stabilising globular proteins and amyloid fibrils and that such differences can be described by giving different weights to hydrophobicity and hydrogen bonding interactions in the two states. In this view, hydrophobicity and hydrogen bonding do not represent fundamental interactions but effective ones, which result from constrained sampling procedures such as those defined by Eq. [2].

### Two-body terms

We decomposed the MJ and PASTA matrices into a combination of the HP (Hydrophobic-Polar) model [11] and a backbone hydrogen bonding model in which all amino acids, except for proline, are capable of forming backbone hydrogen bonds (by analogy, we term this the HB model). These two-body interactions are described by three 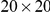 interaction matrices, 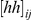, 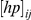 and 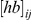, with the following properties: 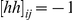 if 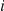 and 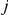 are both hydrophobic residues and topological neighbours, and 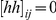 otherwise; 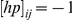 if either 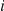 or 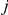 is a hydrophobic residue, 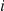 and 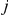 are topological neighbours, and 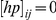 otherwise; 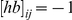 if 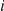 and 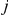 can both form backbone hydrogen bonds and are topological neighbours, otherwise 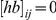.

As a first approximation, we initially fit the MJ and PASTA matrices to an equation of the form:
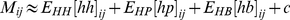
(3)where 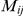 is the matrix of interest, 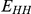, 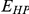 and 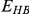 are the weightings of the 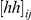, 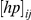 and 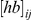 matrices, respectively, and 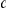 is a constant (the solvent-solvent interaction parameter) [8]. The normalisation constant 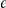 shifts the elements of the MJ and PASTA matrices along the free energy axis thus allowing comparison of 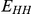, 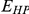 and 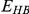 between different matrices. It is used to set the free energy of forming a polar-polar contact, 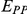, to zero and all other weightings are measured relative to this reference, i.e. 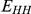 and 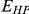 measure the additional free energy of forming hydrophobic contacts and 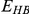 the free energy gained through hydrogen bond formation. Importantly, the adjustment of 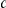 to give 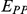 a non-zero free energy has no effect on the ratios of 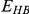 to 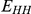 listed in Table 1. The 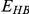 weightings (Table 1) should be, and are, approximately equal to the free energy of a single hydrogen bond (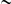
2.5 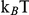 [20]). This simple decomposition given by Eq. [3] gives very good agreement with the MJ (correlation coefficient 0.87) and parallel matrices (correlation coefficient 0.77) and good agreement with the antiparallel matrix (correlation coefficient 0.69, or 0.70 if disulfide bonds are taken into account).

**Table 1:** Hydrophobicity and hydrogen bonding terms (in 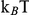) in the HP-HB-SS model.

	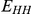	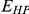	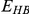	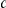	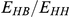
MJ (Native)	3.64 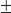 0.10	1.48 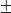 0.09	1.76 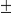 0.12	0.07 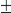 0.14	0.48 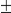 0.10
Parallel (Fibrillar)	1.40 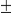 0.09	0.21 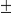 0.08	2.23 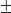 0.11	2.97 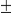 0.12	1.59 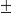 0.13
Antiparallel (Fibrillar)	0.98 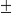 0.08	0.14 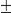 0.07	1.36 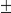 0.09	1.67 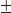 0.11	1.39 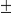 0.15

This coarse-grained HP-HB model is therefore a good approximation to the original matrices, and can thus provide insight into the relative importance of the hydrophobicity and hydrogen bonding terms for the different types of structures (Table 1). Since 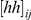, 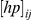 and 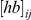 are all binary matrices, it is straightforward to quantify the marginal effect of each of the regressors in our general linear model from the values of their coefficients 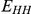, 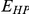 and 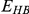.

For the MJ matrix, the ratio of 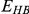 to 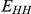 is 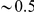 (Table 1) indicating that for protein folding the hydrophobic term is twice as important as the hydrogen bonding term. This ratio was corroborated by decomposing three recent pairwise contact potentials for the native states of globular proteins [21]–[23] which gave a similar result (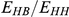 values are 0.4 [21], 0.7 [22], 0.73 [23] and 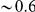 on average). This finding is in agreement with previous work suggesting that the HP model captures the essence of protein folding [11]. Nevertheless, hydrogen bonding does play an important role in protein folding since highly polar sequences can fold to form 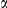-helices, and “side-chain only” molecular dynamics simulations fail to capture crucial aspects of protein folding [24]. Indeed, protein folding simulations have shown that it is necessary to include a mainchain-mainchain hydrogen bonding term in order to obtain secondary structure [25].

For protein misfolding and amyloid formation, the ratio of 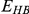 to 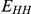 for both PASTA matrices is 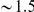 (Table 1) suggesting that backbone-only hydrogen bonding is about 50% more important in stabilising amyloid fibrils than hydrophobic interactions. To demonstrate the robustness of this result, we tested the sensitivity of the 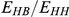 ratio to the Pro-X elements of the PASTA matrices and calculated that the high values of the Pro-X side-chain interaction free energies in the parallel and antiparallel matrices would have to be reduced by 4 or 5-fold respectively to achieve the same ratio of 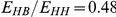 found in the MJ matrix. Given that the side-chain interaction free energies are derived from the Boltzmann relation 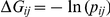, and that the high Pro-X interaction free energies reflect the infrequent occurrence of proline residues in 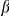-sheets, a reduction of this magnitude would translate into a much greater number of Pro-X contacts being detected in the 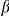-sheets of the PDB dataset used by the authors of PASTA [9]. The increased weighting of the 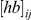 matrix relative to the 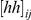 matrix in the decomposition of the PASTA matrices shows that the destabilising effect of proline is more disruptive to the hydrogen bonded 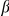-sheet structure than to the native fold of globular proteins in which proline has evolved to play an important structural, and stabilising, role e.g. in Pro-induced 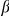-turns [26]. This result underscores the importance of sequence-independent hydrogen bonding in defining the amyloid structure. This “generic” view [12] is consistent with the observation that even hydrophilic and homopolymeric sequences of amino acids can form amyloid fibrils [13]. However, the amino acid sequences of individual peptides and proteins influence their specific propensity to aggregate [16], [17], and to form self-complementary side-chain packing interfaces between adjacent 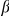-sheets in the fibrils [15], [27], [28]. We also note that in the 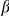-sheets of globular proteins, the effects of backbone hydrogen bonding tends to be averaged out in Eq. (2) by the presence of other secondary structure motifs (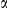-helices, 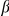-turns and coil).

A number of controls were performed to confirm that the ratio of 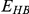 to 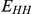 is inverted between folded globular proteins and amyloid fibrils. Firstly, the value of 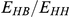 is only slightly affected by considering amino acids such as Proline and Alanine to be hydrophilic rather than hydrophobic. In our initial classification of hydrophobic and hydrophilic residues [11], the ratios between the hydrogen bonding and hydrophobic terms, 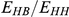, are 0.48, 1.59 and 1.39 for the MJ, parallel and antiparallel PASTA matrices respectively (Table 1). By considering proline residues to be hydrophilic, rather than hydrophobic, the ratios 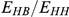 become 0.55, 1.78 and 1.66 for the MJ, parallel and antiparallel PASTA matrices respectively. Furthermore, if we adopt the partitioning suggested by Li, et al. [6] in which both proline and alanine residues are considered to be hydrophilic rather than hydrophobic, the ratios 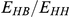 become 0.61, 2.14 and 2.27 for the MJ, parallel and antiparallel PASTA matrices respectively. This analysis shows that the ratio 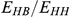 is inverted between the MJ and PASTA matrices using the most common classifications of amino acids into hydrophilic and hydrophobic sets.

We also note that the MJ matrix is calculated by using the quasi-chemical approximation in which protein residues are assumed to be in equilibrium with the solvent. By considering water to be the reference state, all residue-residue interactions are attractive and so all elements of the MJ matrix are negative. By ignoring chain connectivity, it has been argued that this “connectivity effect” introduces a bias into the MJ matrix. However, a knowledge-based pair potential for describing amino acid interactions in the native folds of globular proteins developed by Skolnick, et al. [21], which we refer to as the SJKG matrix, explicitly includes effects due to chain connectivity. Skolnick, et al. [21] conclude that ignoring chain connectivity does not introduce errors and that the quasi-chemical approximation is sufficient for extracting statistical potentials such as the MJ matrix. By virtue of using native reference states, the SJKG matrix has both positive and negative side-chain interaction free energies and is similar in this way to the PASTA matrices (Fig. 1B,C). The SJKG matrix also has a mean free energy of approximately zero (0.08 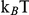) like the PASTA matrices (0.51 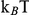 and 0.13 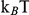 for parallel and antiparallel respectively, Fig. 1B,C). However, like the MJ matrix, the SJKG is a statistical potential for the native folds of globular proteins and when we decompose this matrix using the HP-HB model we get a ratio of 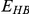 to 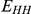 of 0.4, which is almost identical to the ratio 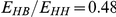 found for the MJ matrix. Thus, this result strengthens our findings as the hydrophobicity term, 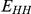, is even more dominant than the hydrogen bonding term, 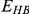, in the decomposition of the SJKG matrix than in the MJ matrix (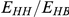 ratios of 2.50 and 2.08 respectively). In addition, the comparison of the value of the normalisation constant 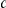 (0.94 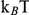) with the values of the 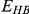 and 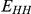 terms (0.49 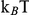 and 1.24 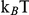, respectively) in the HP-HB decomposition of the SJKG matrix confirms that the value of 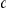 does not affect the ratio of 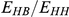 for native proteins and that this ratio is reversed between folded globular proteins and amyloid fibrils.

From the contour maps (Fig. 1A,B,C) and the histograms of contact free energies (Fig. 1D,E,F) it is clear that the free energy of forming hydrophobic-polar (H-P) side-chain contacts is stabilising for globular proteins although not nearly as important in the simple formation of 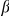-sheets. Thus, for protein folding we find that 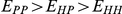 where 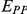 is the free energy of forming a polar-polar contact and is not stabilising (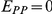) and 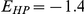 and 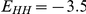 are the free energies of forming hydrophobic-polar contacts and hydrophobic-hydrophobic contacts respectively. These weightings are in excellent agreement with a modified form of the HP model [29] (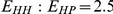 in the present study compared to 2.3 in the modified HP model [29]) and so validate its use in protein folding simulations.

The inclusion of the HP term in Eq. [3] has only a marginal effect on the regression to the parallel or antiparallel matrices as demonstrated by the relatively small coefficient 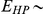
0.2 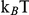 (Table 1). This result suggests that the segregation of hydrophobic and polar residues is not very important in 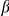-sheet formation and could lead to solvent exposed non-polar side-chains in prefibrillar aggregates, a feature that has been suggested to be closely linked to cytotoxicity [30]. The minor effect of the HP term is also in accord with our finding that hydrophobic interactions play a less significant role than inter-molecular hydrogen bonding in stabilising amyloid fibrils and again supports the idea that peptides and proteins are prone to forming amyloid structures irrespective of sequence [12], [13], although the relative propensities to form such structures will vary with sequence [16], [27].

### One-body terms

**Figure 2:**
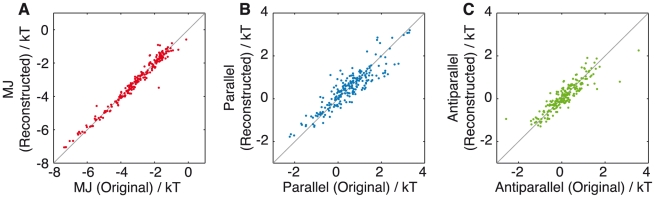
Correlation between the original matrix elements and the matrix elements reconstructed from **equation (4**). (A) MJ matrix, 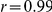, rmsd 0.23 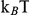. (B) Parallel matrix, 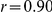, rmsd 0.42 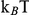. (C) Antiparallel matrix, 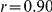, rmsd 0.32 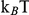.

Previous analyses of the MJ matrix shows that two-body interactions are not sufficient to capture all of the details of the 210 independent amino acid interactions that describe the variety of native protein structures [6]–[8]. A one-body term, 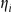, describing the individual properties of each amino acid, is also required. Adding this additional term to our previous free energy expression Eq.[**3**] gives
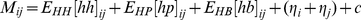
(4)The application of this equation to the MJ, parallel and antiparallel matrices gives correlation coefficients of 0.99, 0.90 and 0.90 respectively (Fig. 2A,B,C). This expression, therefore, describes the original data extremely well and suggests that the diverse and complex interactions stabilising both the native and fibrillar states are amenable to a low-dimensional representation using simple two-body and one-body terms [6]–[8].

It is remarkable that the same approach can be used to decompose both the MJ and PASTA matrices, indicating that the underlying interactions are the same but that the balance is different, and leads to a clear demarcation of the thermodynamic minima of the native and amyloid states of the protein free energy landscape.

**Figure 3:**
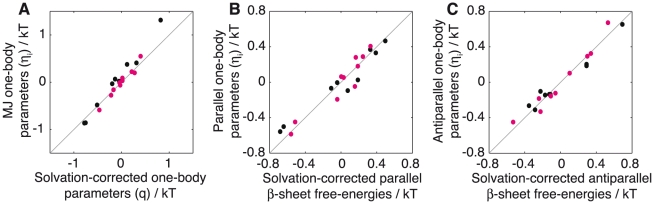
Correlation between the solvation-corrected free energy of secondary structure formation and one-body parameters 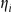. (A) Solvation-corrected one-body parameters 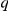 *vs* MJ one-body parameters 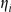, (B) Solvation-corrected parallel 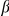-sheet free energies *vs* parallel one-body parameters 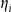, and (C) correlation between the solvation-corrected antiparallel 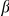-sheet free energies and the antiparallel one-body parameters 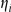. Hydrophobic residues are shown in black and hydrophilic residues in magenta. Correlation coefficients are 0.98, 0.96 and 0.97, respectively, and the root mean square deviations are 0.16, 0.10 and 0.07 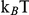 respectively.

The three sets of 20 one-body parameters, 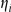, that are derived from the MJ, parallel and antiparallel matrices are listed in Table 2. Previous work has shown that one-body components of the MJ matrix, known as q-values, are closely related to the interactions governing secondary structure formation [6]. We find that our equivalent one-body potentials, MJ 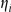 (Table 2), correlate extremely well with (correlation coefficient of 0.98, Fig. 3A), and are numerically almost identical to this previously published q-scale (Table 2, column 4) provided that the hydrophobic and hydrophilic q-values are separated and have their respective mean values subtracted from each non-polar and polar element. This procedure removes an average hydrophobic penalty for non-polar residues (+1.45 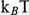) and an average hydrophilic gain for polar residues (−0.07 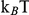). This residue-specific hydrophobic (hydrophilic) cost (gain) can be interpreted as an average free energy cost of placing in water the surface of a given residue plus the gain of attractive dipolar interaction between the residue concerned and water, with polar residues being more favourable than non-polar residues [7].

**Table 2:** One-body potentials, 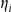, for the matrices for the MJ (native) case, the parallel fibril case and the antiparallel fibril case in the HP-HB-SS model, and free energies for secondary structure formation, 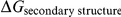, in 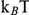 [6], [31]. 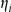 corresponds to the sum of the free energy of formation of secondary structure, 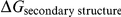 and the free energy of solvation, 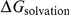 (Eq. [5]).

	MJ	Parallel	Antiparallel	q-values [6]	Parallel 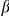 -sheet	Antiparallel 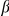 -sheet
	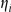	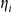	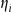		free energy [31]	free energy [31]
C	0.3775	0.3314	−0.1364	−1.3330	0.0685	−0.4569
F	−0.8575	−0.0677	−0.1439	−2.2031	−0.4304	−0.5163
L	−0.8635	−0.0037	0.2036	−2.2283	−0.3633	−0.0535
W	0.0220	0.3693	−0.1354	−1.4989	0.0069	−0.4696
V	0.0665	−0.5571	−0.2639	−1.5845	−1.0009	−0.6972
I	−0.4815	−0.5002	−0.1024	−1.9617	−0.9620	−0.5686
M	−0.0320	0.0258	0.1861	−1.6448	−0.1320	−0.0535
Y	0.4090	−0.0946	−0.3104	−1.1368	−0.2450	−0.6292
A	1.3140	0.4663	0.6531	−0.6288	0.1752	0.3474
P	0.0455	0.0304	0.0496	−0.2716	1.3643	1.0544
H	−0.5874	−0.4483	−0.3311	−0.5382	0.0008	0.0305
G	−0.1594	0.0632	0.2939	−0.2414	0.5167	0.5544
N	0.0891	0.1812	0.3249	−0.0553	0.7016	0.5942
T	−0.2749	−0.5853	−0.4491	−0.2917	−0.0449	−0.2755
S	0.0316	−0.1928	−0.1561	−0.0553	0.4718	0.1457
R	−0.0624	0.0532	−0.1831	−0.1006	0.5432	0.0133
Q	−0.0094	−0.0473	−0.1226	−0.1157	0.6694	0.1976
D	0.1986	0.4062	0.6719	0.2012	0.8437	0.7852
K	0.5506	0.2807	−0.1516	0.3270	0.6792	0.1447
E	0.2236	0.2892	0.1029	0.1408	0.7587	0.3565

This effect is even more apparent in the simpler case of the one-body components of the parallel and antiparallel PASTA matrices (Table 2, parallel 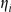 and antiparallel 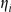 respectively). When existing parallel and antiparallel 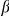-sheet propensity scales [31] are converted into free energies (Table 2, column 5 and 6 respectively), grouped into polar and non-polar terms and then separately shifted to have zero mean, thus removing the average hydrophobic (hydrophilic) cost (gain) to water of forming a 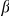 sheet (the values are +0.32 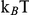 (−0.51 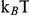) and +0.34 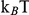 (−0.25 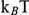) for parallel and antiparallel 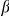-sheets respectively), the remainder correlates extremely well with (correlation coefficients of 0.96 and 0.97 for parallel and antiparallel 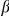-sheets respectively, Fig. 3B, C), and is numerically almost identical to the one-body potentials of the parallel and antiparallel matrices (parallel 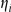 and antiparallel 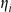 respectively, Table 2). This result suggests that the one-body free energy components of the MJ, parallel and antiparallel matrices are given by
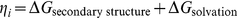
(5)where 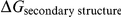 represents the free energy to form hydrogen bonded secondary structure and 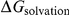 is an average free energy of solvation. Hence, we suggest that the one-body free energy terms, 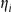, correspond to a stabilisation of the native or fibrillar state through a competition between hydrophilicity and the formation of hydrogen bonded secondary structure.

### Hydrophobicity and hydrogen bonding sculpt the free energy landscape of a protein

The HP-HB-SS (HP-HB-secondary structure) model described above suggests therefore that both the globular and amyloid states of proteins are stabilised by hydrophobic interactions, hydrogen bonding and the formation of secondary structure, and that there is a common form for the effective Hamiltonian, 
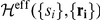
, describing both protein folding and misfolding, given by the substitution of Eq.[**4**] into Eq.[**1**]
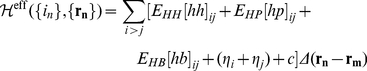
(6)The two-body terms in the effective Hamiltonian are 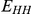, 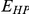 and 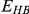, which correspond to the relative strengths of hydrophobic interactions and hydrogen bonding, and take the values given in Table 1. The effective energy function is further modulated by the additive residue specific 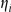 terms (Table 2), which correspond to the free energy of secondary structure formation plus a free energy of solvation. It is important to note that there is a loss of translational and rotational entropy on going from native to fibrillar states [32] which we do not consider here. This loss of entropy would be expected to stabilise the native state in a sequence- and conformation-independent manner and would add a native-biasing term to the effective energy function given in Eq. [6].

Although the general form of the effective Hamiltonian is the same for protein folding and misfolding, the variables 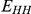, 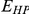, 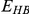 and 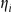 are different for these two processes, with the result that the minima in the two cases will occur at different positions in conformational space. Fibrillar aggregates represent a well-defined region of the wider protein folding landscape characterised by the pervasiveness of generic intermolecular hydrogen bonding [12]. Since the Hamiltonian maps the sequence space on to the structure space, as the weights 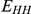, 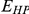 and 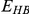 change so too does the shape of the resulting structure. The dominance of the collapse-inducing hydrophobic force in protein folding leads to a globular tertiary structure, with hydrophobic residues buried in the core and largely polar residues on the surface of the protein [33]. However, when unidirectional inter-molecular hydrogen bonding is in the ascendancy, the result is ordered protein self-association into elongated, rigid, rod-like aggregates [14].

### Local vs non-local effects

By decomposing the MJ and PASTA matrices into two-body and one-body components, we have effectively decoupled the two-body non-local interactions from the one-body, local interactions entangled in these statistical potentials. This approach enables us to analyse quantitatively the relative importance of local and non-local interactions in determining the folding and misfolding of proteins. It is clear from Tables 1 and 2 that the magnitude of the non-local (tertiary) interactions are significantly greater than the local (secondary) interactions in stabilising the native protein or fibrillar aggregate. This result indicates that nonlocal inter-residue interactions are the major determinant of secondary structure in the HP-HB-SS model. This finding is in excellent agreement with a large body of experimental [34] and computational analyses [35], which demonstrates that the sequence patterns of polar and non-polar amino acids dominate their intrinsic secondary structure propensities in determining the secondary structure motifs of a globular protein [36] or amyloid fibril [37]. Our prediction that hydrophobic patterning and sequence independent hydrogen bonding is more important than residue-specific identity in shaping secondary and tertiary structure helps explain why a wide variety of amino acid sequences can encode the same basic protein fold [38]. It is also consistent with the mutational robustness of functional proteins, which typically only fail to fold correctly following several mutations of individual amino acids [39]. In addition, globular proteins have evolved to mitigate against the non-local effect of polar/nonpolar periodicity by deliberately spurning alternating hydrophobic patterns which program amino acid sequences to form amphiphilic 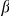-sheets and amyloid fibrils [40]. This is further evidence that tertiary interactions overwhelm the intrinsic propensities of individual amino acids in real proteins, which agrees with our analysis.

### Role of frustration in defining the protein free energy landscape

The mathematical form of the effective Hamiltonian of Eq. [6] describing protein folding and misfolding is analogous to that of a spin glass model in which competition between conflicting interactions leads to a rugged free energy landscape [41]. Apart from topological frustration, which arises due to chain connectivity, the three sources of energetic frustration in the HP-HB-SS model stem from the competition between intramolecular collapse and intermolecular self-association, the contest between frustrating nonlocal interactions and, finally, the inability to satisfy simultaneously all local secondary structure preferences. As discussed earlier, in our model the relative strengths of the hydrophobicity to hydrogen bonding terms governs the dichotomy between folding and misfolding (Table 1). The conflicting optimisation factors imposed by hydrophobic clustering, maximal backbone hydrogen bonding and the segregation of hydrophobic and polar residues prevent the native state or fibrillar aggregate from energetically satisfying all of these inter-residue interactions. Finally, since non-local interactions predominantly determine globular [36] and fibrillar protein structures [37], there is an additional source of mismatch between the secondary structure motifs encoded by the hydrophobic patterning of the amino acid sequence as a whole and the secondary structure propensities of the individual amino acids.

This intricate interplay of competing interactions gives rise to multiple local minima in the effective energy function of Eq. [6] but, in accordance with the principle of minimal frustration [2], the sequence of a protein has evolved to reduce the number of alternative minima as much as possible and to have its native state as the global minimum of the protein folding free energy landscape [2], [3]. However, the ruggedness of the folding free energy landscape increases the likelihood that excited native-like states exist, which may be transiently populated *via* thermal fluctuations, thus potentially leading to amyloid formation even under physiological conditions [42]. Moreover, frustration in the protein misfolding free energy landscape can lead to amyloid fibril polymorphs with different physical and biological properties [43].

Lowering the discordance between non-local (Table 1) and local (Table 2) interactions leads to more stable and cooperative native protein folds [35], [44], and has implications for the *de novo* design of proteins [44] and amyloid fibrils [45], [46]. Indeed, knowledge of the residue-specific one-body terms (Table 2), and the understanding that they correspond to the free energy of secondary structure formation once a solvation free energy is taken into account, may aid in the rational design of globular folds through mutational screening of regions known to be critical for aggregation.

## Discussion

The present work indicates that there are common intermolecular forces stabilizing both globular and fibrillar states of proteins, but that a different balance of these forces results in either folding or misfolding to non-functional and potentially toxic aggregates. This situation occurs as the competing processes of protein folding and misfolding are finely tuned in terms of their free energies. Upon folding, the protein minimises the free energy of the protein-water system by clustering hydrophobic groups and forming intramolecular hydrogen bonds in the globular interior. By contrast, upon aggregation into amyloid fibrils, the formation of an extensive intermolecular hydrogen bonding network compensates for any exposure of hydrophobic groups to water that results from the fibrillar structure of the aggregated state.

It has been found in molecular dynamics simulations that the correct balance between hydrophobicity and hydrogen bonding must be attained for proteins to fold correctly or to self-assemble into the alternative well-defined amyloid structure rather than into amorphous aggregates [19], [47]. For example, if hydrophobicity is too dominant, then an amorphous cluster of residues with few native contacts can be formed rather than a correctly folded protein [19]. Interestingly, these simulations suggest that hydrogen bonding is more than twice as important as hydrophobicity for aggregation into amyloid fibrils [19], [48], and that hydrophobicity is approximately twice as important as hydrogen bonding for protein folding [19], findings that are in close agreement with those reported by the analysis in the present paper. Recent experimental evidence supports this interpretation of protein folding and misfolding. It has been found that the substitution of backbone ester groups for the amide linkage does not significantly affect the structure of native proteins [49], suggesting that the folded core is mainly stabilised by hydrophobic interactions. Similar experiments for protein aggregation, however, reveal that peptides with removed backbone amide groups have a much reduced propensity to form ordered aggregates [50]; indeed such species are being explored as potential therapeutic inhibitors of amyloid fibril growth [51]. In addition, the large elastic modulus of amyloid fibrils stems mainly from generic inter-backbone hydrogen bonding indicating that this is a dominant interaction defining the amyloid state [14].

The weights 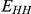, 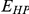 and 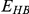 are functions of physical [52], [53] and chemical [54]–[56] parameters. Hydrophobic attraction, 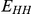, and hydrogen bond interaction strength, 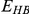, are both strongly environment-dependent intermolecular forces and vary in a complex manner as externally driven parameters such as temperature, pH, ionic strength and denaturant concentration are changed [32]. Despite the complicated nature of these interactions, experiments show that at low concentration, denaturants increase the monomer-monomer dissociation energy approximately linearly [54]. This suggests that the monomer-monomer association energy 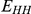 is a linear decreasing function of denaturant concentration under mildly denaturing conditions. In keeping with our model, we speculate that at low denaturant concentrations, 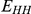 is large, thereby promoting the native state by increasing residue-residue hydrophobic attraction, whereas at higher denaturant concentrations the lowering of 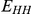 leads to destabilisation of the hydrophobic core of the native structure, making intermolecular association much more likely [57]. Our analysis suggests that there is an optimal balance between hydrophobicity and hydrogen bonding for protein folding and a significant redistribution of these intermolecular forces for amyloid formation. Such a shift in balance can be seen as a jump between free energy landscape minima, and could occur, for example, at a critical concentration [58], or pH [55], or at a temperature sufficiently high to overcome kinetic barriers between the native and amyloid minima [46]. Overall, however, this balance appears to be very finely tuned for both protein folding and misfolding, and it is interesting to speculate on the role of this delicate balance of forces within the cell.

It has been suggested that proteins have evolved to be expressed intra-cellularly at levels in the region of the critical concentration for aggregation [58]. While a plentiful abundance of a given protein in the cell optimises its function, being on the verge of insolubility leaves proteins susceptible to environmental changes and prone to aggregation [59]. Our findings are consistent with this hypothesis [58], since elevated protein levels increase the likelihood of intermolecular as opposed to intramolecular interactions, and suggest that a precarious balance between hydrogen bonding and hydrophobic forces dictates whether peptides and proteins adopt normal or aberrant biological roles.

In conclusion, we have reported an interpretation of statistical potentials for protein folding [5] and misfolding [9] by expressing them in terms of a model containing specific terms for hydrogen bonding and hydrophobicity. This approach has enabled us to describe complex and diverse interactions using specific values of three distinct two-body terms and intrinsic secondary structure propensities. We have explained the significance of each of these terms and derived a physically meaningful common form of effective Hamiltonian for both protein folding and amyloid formation. This approach suggests that while hydrophobicity, hydrogen bonding and the formation of secondary structure are important to both processes, the balance between hydrophobicity and hydrogen bonding is remarkably different in the two regimes. Our central finding is that the stabilities of correctly folded proteins are dominated by side-chain hydrophobic interactions and that amyloid fibrils are stabilised mainly by sequence-independent intermolecular hydrogen bonding. We have also quantified the relative importance of local and non-local interactions in determining the structure and stability of proteins in both their globular and fibrillar forms and find that inter-residue interactions are more influential than secondary structure propensities in shaping the final native or amyloid fold. This result shows that, in accordance with the principle of minimal frustration [2], natural proteins have evolved to maintain a low ratio of local-to-non-local interaction strengths, thereby minimising the effect of a potent source of frustration and ensuring cooperative and stable folding [35], [44].

In summary, we have found that the conflict between protein folding and misfolding is governed by the contest between a side-chain-driven hydrophobic collapse and a backbone-driven self-association. The almost infinite variety of outcomes of such a conflict gives rise to the rich and diverse behaviour exhibited by proteins and the resulting balance between health and disease.

## Methods

### Two-body terms

The weights of the two-body terms, 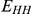, 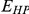, 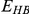, and the constant, 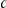, were determined by performing multiple regression in MATLAB.

### One-body terms

The twenty one-body terms, 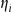, were determined by performing a simulated annealing minimisation in MATLAB.
